# The application of ERAS in pilonidal sinus: comparison of postoperative recovery between primary suture and Limberg flap procedure in a multicenter prospective randomized trial

**DOI:** 10.3389/fsurg.2024.1120923

**Published:** 2024-06-13

**Authors:** Wei Lu, Shujuan Huang, Hui Ye, Shang Xiang, Xiangsheng Zeng

**Affiliations:** ^1^ Department of Colorectal and Anal Surgery, Taizhou Affiliated Hospital of Nanjing University of Traditional Chinese Medicine, Taizhou, China; ^2^ Department of Colorectal and Anal Surgery, Jingzhou Hospital Affiliated to Yangtze University, Jingzhou, China; ^3^ Department of Neurosurgery, Taizhou Affiliated Hospital of Nanjing University of Traditional Chinese Medicine, Taizhou, China; ^4^ Department of Respiratory and Critical Care Medicine, Jingzhou Hospital Affiliated to Yangtze University, Jingzhou, China

**Keywords:** Limberg flap, rhomboid flap, pilonidal sinus, enhanced recovery after surgery, surgery

## Abstract

**Purpose:**

We evaluated the clinical effect of utilizing a Limberg rhomboid flap graft in conjunction with Enhanced Recovery After Surgery (ERAS) protocols for the management of pilonidal sinus in the sacrococcygeal region to demonstrate the feasibility of applying ERAS to the treatment of pilonidal sinus.

**Methods:**

Between January 2010 and August 2018, prospective data analysis was undertaken on 109 patients who received surgical treatment for pilonidal sinus in the sacrococcygeal region at the Department of Colorectal and Anal Surgery, Jingzhou Hospital affiliated to Yangtze University, and Taizhou Affiliated Hospital of Nanjing University of Chinese Medicine. The patients were randomly separated into two groups based onoperation technique: the control group (pilonidal sinus resection with primary suture) and the observation group (pilonidal sinus resection with Limberg flap graft). Some patients in the above two groups received ERAS after surgery, which included early feeding and early ambulation, etc. Therefore, we further subdivided each group into group A (without ERAS) and group B (with ERAS) according to whether they received ERAS. Comparative analysis was conducted to assess differences in pertinent data before and after surgery across the respective groups.

**Results:**

The length of postoperative hospitalization was shorter and wound dehiscence was more common in control group B than in control group A [(9.00 ± 1.20) vs. (11.07 ± 1.78), 26.7% (8/30) vs. 7.1% (2/28), *P* < 0.05]. Observation group B exhibited significantly shorter wound recovery periods and postoperative hospital stays compared to observation group A [(8.08 ± 1.20) vs. (9.16 ± 2.21), (26.23 ± 3.97) vs. (29.08 ± 4.74), *P* < 0.05]. The hospitalization duration and wound healing time in observation group B were notably shorter than those observed in control group B [(8.08 ± 1.20) vs. (9.00 ± 1.20), [26.23 ± 3.97 vs. (43.67 ± 7.26), *P* < 0.05], but the operation time was longer and scar acceptance was lower [(78.85 ± 10.16) vs. (43.30 ± 6.06), (4.00 ± 0.69) vs. (7.53 ± 0.86), *P* < 0.05]. The VAS score, infection rate, wound dehiscence rate, subcutaneous hematoma rate and 5-year recurrence rate in observation group B were lower than those in control group B [(5.00 ± 1.39) vs. (7.13 ± 0.78), 3.8% (1/26) vs. 23.3% (7/30), 3.8% (1/26) vs. 26.7% (8/30), 3.8% (1/26) vs. 26.7%(8/30), 7.7% (2/26) vs. 30.0% (9/30), *P* < 0.05], but the rate of flap ischemia or necrosis was higher [15.4% (4/26) vs. 0(0/30), *P* < 0.05].

**Conclusion:**

The combination of ERAS with pilonidal sinus resection using Limberg flap graft demonstrated a reduction in infection rates, wound dehiscence, subcutaneous hematoma occurrence, and recurrence rates, along with alleviation of postoperative pain and acceleration of healing time. Comparatively, this approach offers superior advantages over pilonidal sinus resection with primary suture in the management of sacrococcygeal pilonidal sinus.

## 1 Introduction

Herbert Mayo was the first to describe a cystic disease affecting the subcutaneous hair in the sacrococcygeal region in 1,833 (1). Subsequently, in 1880, Hodges officially termed this condition “pilonidal sinus” (2). It was later termed “Jeep disease” because of the high incidence of driving jeeps in the U.S. Army during World War II (3). The pilonidal sinus is an infectious sinus tract situated subcutaneously in the sacrococcygeal region. It typically originates in the gluteal sulcus and extends along the gluteal midline. This condition often manifests with chronic intermittent episodes and may also exhibit acute abscess changes, posing challenges for spontaneous healing (4).

Key risk factors contributing to the development of this condition include excessive hairiness, skin damage, inadequate hygiene practices, deep gluteal sulci, prolonged periods of sedentary lifestyle, obesity, and localized skin moisture (5–7). The pathogenesis of the disease is still unclear, with prominent theories including: 1. “Congenital theory”: Proposed by Tourmeaux, this theory posits that the pilonidal sinus in the sacrococcygeal region results from skin depression in the intergluteal fissure, which may stem from a developmental anomaly or congenital malformation in the sacrococcygeal region (8); and 2. “Acquired theory”: This theory suggests that inflammatory reactions arise from blockages within hair follicles or hair penetration into the follicle. It encompasses Karydakis’ foreign body reaction theory (9) and Bascom's hypothesis of “midline concavity” (10, 11). Related studies have indicated that foreign hair can serve as a trigger for sinus formation, as even short, broken hair resulting from haircuts can puncture intact skin (12). Moreover, the most robust hairs found within the sinus are primarily of occipital origin (13). Akinci et al. (14) reported that a deeper natal cleft can increase susceptibility to pilonidal sinus disease. This deeper cleft may facilitate the complete erection of cut hair, thereby exerting significant local force on the skin. In addition, recent studies have shown that sweating may have a protective effect in pilonidal sinus disease rather than being a risk factor (15).

Clinically, the conservative treatment methods for pilonidal sinus of sacrococcygeal region include fistula phenol injection, fibrin glue, laser treatment and radiofrequency ablation (16), but the treatment effect is poor and the recurrence rate is high (17, 18). As such, surgery is the preferred option once pilonidal sinus is diagnosed (19). Surgical treatment primarily encompass incision and drainage, lesion excision with primary suture, lesion excision with flap transfer, among others (20).

Nevertheless, simple incision and drainage often lead to a high recurrence rate, with challenging wound healing, significantly impacting the patient's daily life and work. Lesion excision with primary suture carries the risk of postoperative wound dehiscence due to the heightened tension at the suture site (21). Thus, opting for a surgical method with short hospitalization time, low recurrence rate, fast recovery and few complications can save medical resources. The Limberg flap graft is an ideal treatment for the pilonidal sinus (22). Limberg, a Soviet scholar, proposed a diamond-shaped transfer flap for treating superficial defects in 1946, which was later extensively adopted for the pilonidal sinus, hence the name Limberg flap (23). Flap graft procedures aim to furnish healthy tissue for the coverage of resected lesions. Commonly employed flap graft techniques in clinical practice include the Karydakis flap, Bascom cleft lift, V-Y advancement flap, Z-plasty, among others.

Enhanced recovery after surgery (ERAS) is an initiative that aims to improve the quality of care for surgical patients (24). It achieves this by implementing evidence-based recommendations aimed at optimizing care throughout the preoperative, intraoperative, and postoperative phases (25). The first ERAS guideline was developed for colorectal surgery by Henrik Kehlet and his colleagues from Copenhagen in 1995 (26). Their original set of interventions, supplemented by additional elements, has evolved into what is now recognized as fast-track or ERAS pathways (27). Benefits associated with enhanced recovery pathways include reduced postoperative complications, reduced hospital length of stay, and improved postoperative quality of life as well as reduced overall healthcare costs (28). Early postoperative mobilization is a central tenet of ERAS (24), and several care elements are also adjuvant measures to encourage early mobilization, including those related to optimal postoperative analgesia and early removal of urinary catheters (29). In the present study, the clinical data of 109 patients who underwent pilonidal sinus surgery were analyzed. The patients underwent treatment at the Department of Colorectal and Anal Surgery, Jingzhou Hospital affiliated to Yangtze University, and Taizhou Affiliated Hospital of Nanjing University of Chinese Medicine, from January 2010 to August 2018, including 58 cases of pilonidal sinus resection with primary suture and 51 cases of pilonidal sinus resection with Limberg flap graft. In addition, 30 cases and 26 cases received ERAS, respectively.

## 2 Materials and methods

### 2.1 General information

The study included 80 male and 29 female participants, with ages ranging from 15 to 68 years and a mean age of 27.97 years. The duration of the disease varied from 1 to 39 months, with a mean duration of 11.38 months. Participants had a body mass index (BMI) ranging from 21.22 to 30.22, with a mean BMI of 26.20. The Ferriman-Gallwey score ranged from 2 to 34 points, with a mean score of 13.18 points.

### 2.2 Grouping

According to the different surgical techniques of pilonidal sinus incision, patients were divided into two groups: the control group (pilonidal sinus resection with primary suture) and the observation group (pilonidal sinus resection with Limberg flap graft). The choice of surgical techniques was randomized: cards containing the procedure were placed in two separate envelopes, which were sealed and scrambled, and each new patient entering the study selected and opened one of the envelope. All patients signed an informed consent form for surgery. According to whether they received ERAS, we further subdivided each group into group A (without ERAS) and group B (with ERAS). Patients were also randomly selected to receive ERAS, and the randomized method was the same as the surgical choice.

### 2.3 Case inclusion and exclusion criteria

#### 2.3.1 Inclusion criteria

Inclusion criteria for the study involved adhering to the clinical practice guidelines outlined by the American Society of Colorectal and Rectal Surgeons in their 2019 edition, specifically regarding the diagnosis and treatment of pilonidal sinus, and possessing complete medical records.

#### 2.3.2 Exclusion criteria

The exclusion criteria for the study encompassed: (1) patients with severe immune system disorders; (2) patients intolerant to surgical procedures; (3) patients presenting with significant anemia, hypoproteinemia, or other conditions that may impede incision healing; (4) patients with severe cardiovascular diseases, liver or kidney diseases; (5) pregnant or lactating women; (6) patients with severe metabolic disorders; (7) patients with a history of neurological or psychiatric conditions; and (8) patients with incomplete follow-up data are also excluded from participation.

### 2.4 Surgical method

**Figure 1:**
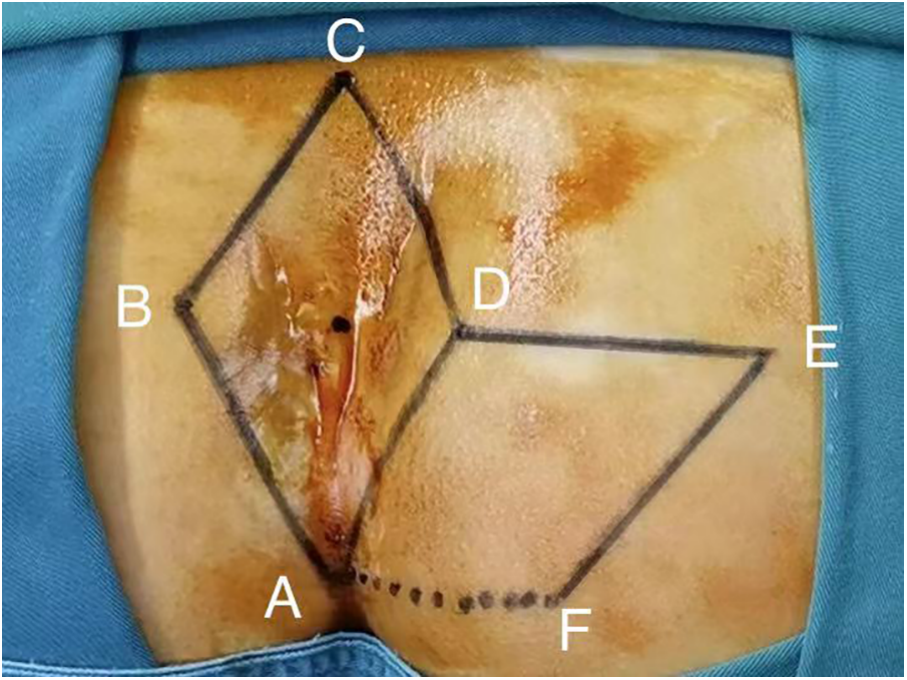
Mark the area for surgery and planned flap.

**Figure 2:**
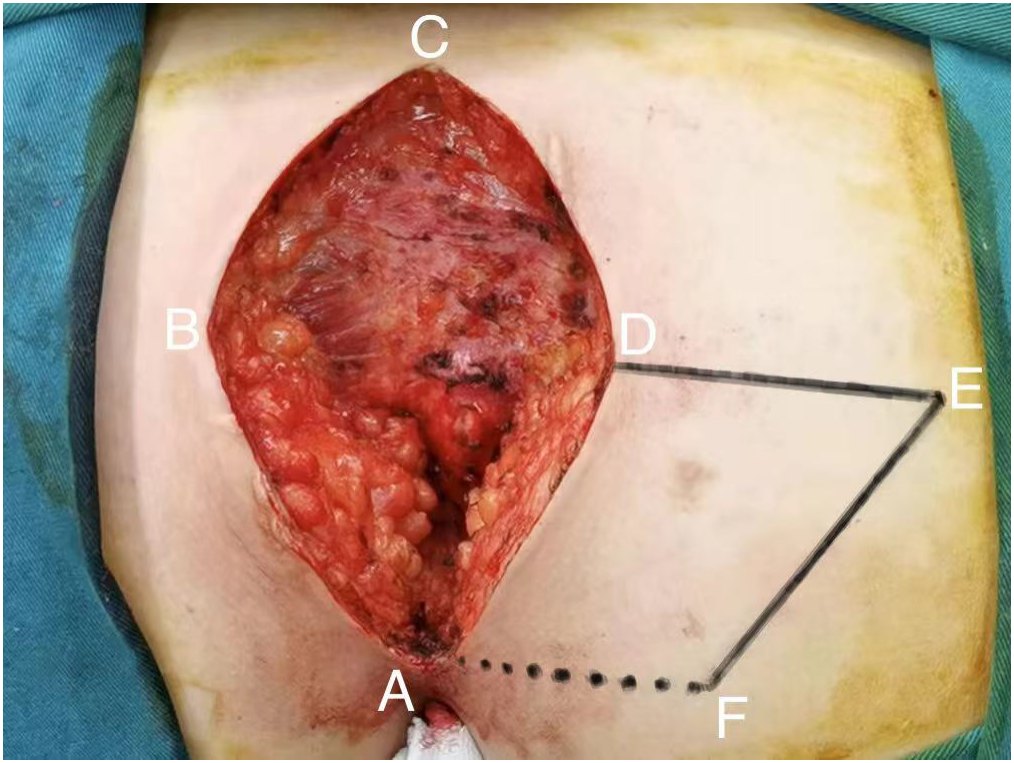
Excision of lesion.

**Figure 3:**
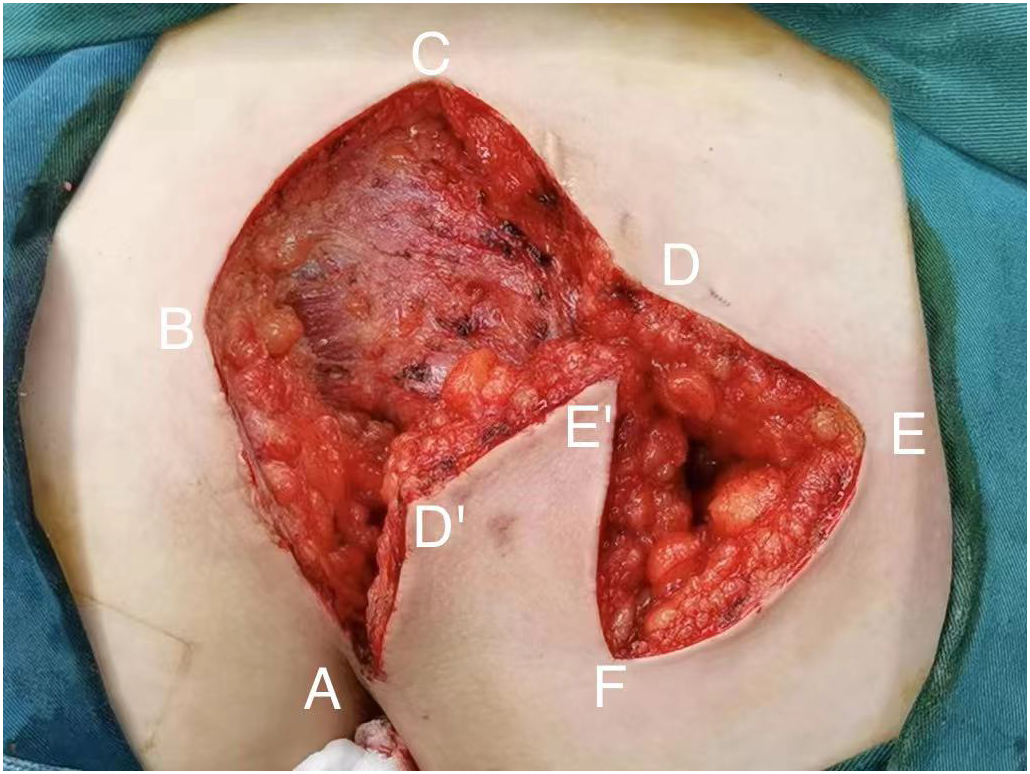
Free skin flap.

**Figure 4:**
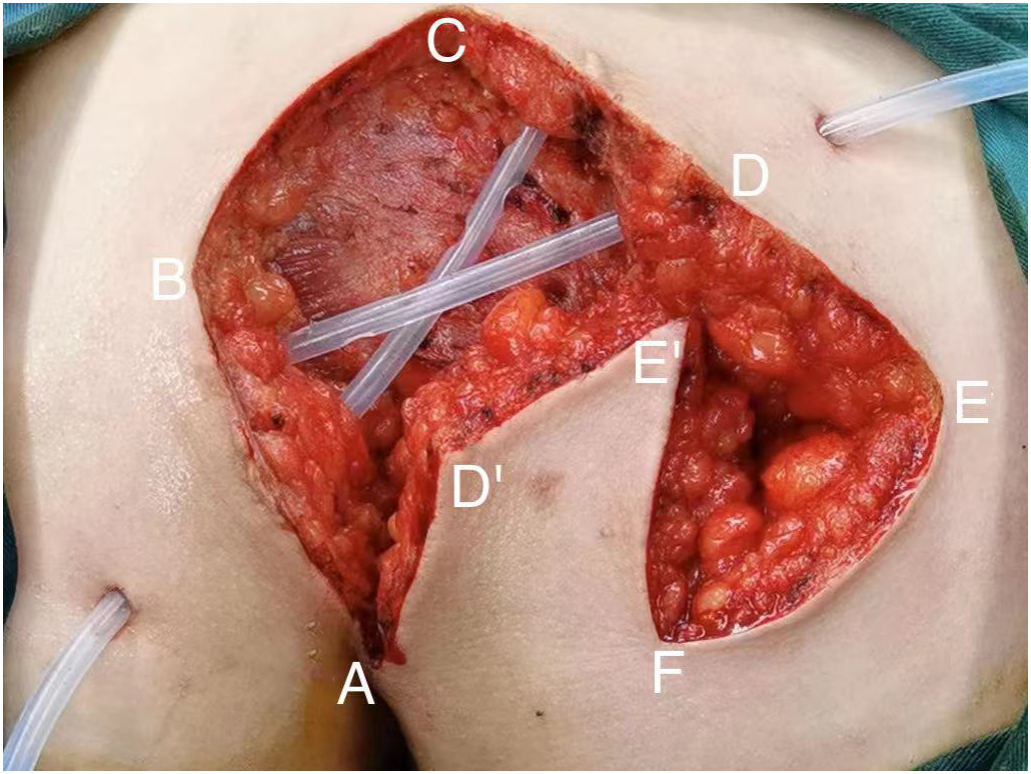
Place two drain tubes.

**Figure 5:**
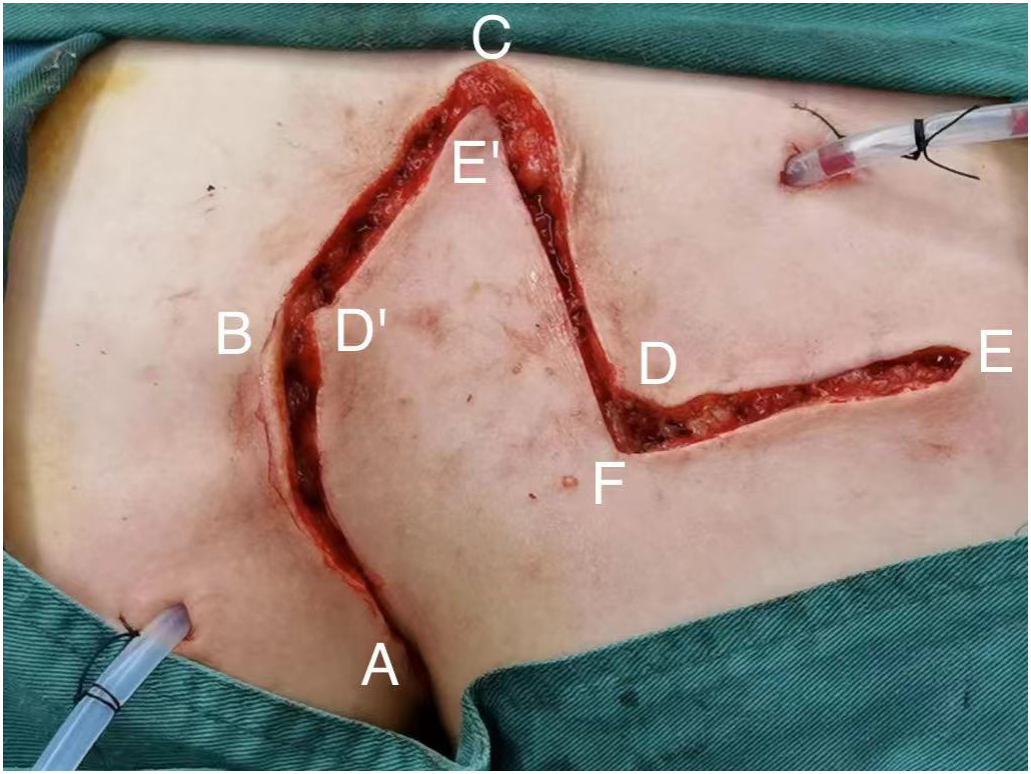
Corresponding angle involution and suture.

**Figure 6:**
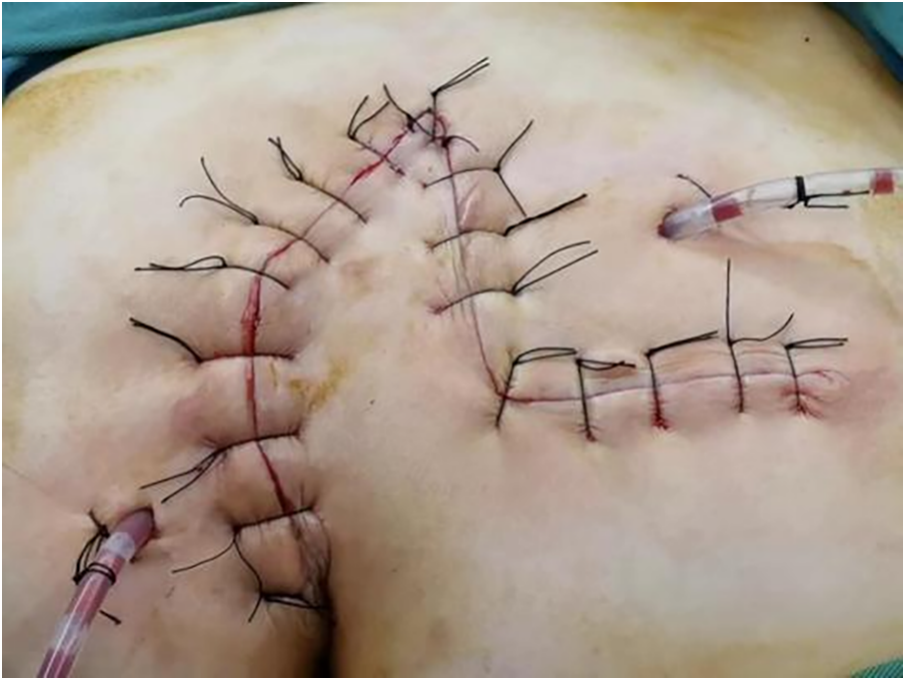
Appearance of sacrococcygeal region after operation.

All patients with pilonidal sinus underwent preoperative enema and skin preparation. The surgical procedure necessitated the presence of at least two proficient surgeons. Anesthesia administration involved combined spinal-epidural anesthesia. Subsequent pathological analysis was conducted on resected specimens following the surgery. (1) Control group: Procedures involved injecting a solution comprising hydrogen peroxide, methylene blue, and saline into the external opening of the sinus. The skin was then incised in a shuttle shape with the sinus opening serving as the central point. Special attention was directed towards tissues stained by methylene blue during lesion excision. The excision range was judiciously increased based on guidance from methylene blue staining and preoperative magnetic resonance imaging of the sacrococcygeal region, with the aim of thoroughly removing the sinus and surrounding infected tissues. Care was taken to avoid damaging the sacrococcygeal bone and its periosteum. Additionally, meticulous hemostasis was ensured throughout the operation. After rinsing the trauma with hydrogen peroxide and a sufficient amount of saline, the sterile gloves and surgical instruments were replaced. A drainage tube was inserted at the wound base after verifying the absence of noticeable blood leakage. Closure of the wound was achieved using 0 Silk sutures, followed by application of sterile dressing and pressure dressing. Subsequently, the drainage tube was connected to a negative pressure system. (2) Observation group: the lesion served as the focal point, with points A, B, C, and D marked around it. These points were sequentially connected to form a rhombus, constituting the surgical resection area, with ∠BCD measuring 60°. Line BD was extended to point E, ensuring that line DE matched the length of the rhombus side. Using a straightedge, line AD was aligned to coincide with point E, forming point F. Points E and F were connected, resulting in the formation of the flap area, comprising points A, D, E, and F (Figure 1). The ABCD rhombic lesion area was entirely excised using an electric knife (Figure 2), removing all involved tissues, including the sinus tract and any concavity in the midline down to the surface of the sacral fascia. The wound was then irrigated with hydrogen peroxide and saline, and sterile gloves and surgical instruments were replaced. Subsequently, the skin and subcutaneous tissues were dissected along DE and EF to fully release the rhombic flap (Figure 3). Two drainage tubes were inserted and secured (Figure 4). The rhombic flap was then transferred, aligning point E’ of the flap with point C of the wound, and the subcutaneous fascia layer was sutured using 2-0 Vicryl. Points D’ and B were treated simultaneously with points D and F, respectively (Figure 5). During suturing, an assistant compressed the area to be stitched toward the center to minimize tension. The fascial layer and skin between the two parts were intermittently sutured (Figure 6). A negative pressure drainage bag was applied, and the wound was dressed with pressure.

### 2.5 Postoperative management

Patients were treated with routine postoperative anti-infection therapy, and received a three-day course of prophylactic antibiotics following the surgical procedure. Negative pressure drainage was utilized to remove fluids and facilitate contact between wound margins. Wound dressings were replaced 3–4 times weekly. Particular care was taken to prevent bending of the back and other movements that could increase tension in the sacrococcygeal area. Stitches could be removed approximately 2 weeks post-surgery, depending on the wound's healing progress. In the conventional rehabilitation group (Group A), early mobilization was discouraged, and the time before the first meal was extended to consequently delay the time of first defecation. The patients were instructed to start eating on the 5th day after surgery and to try to get out of bed on the 7th day. In the Combined ERAS group (Group B), the ERAS principles were implemented in postoperative care. Patients gradually resumed mobility after 48 h of bed rest. We instructed the patient to try to sit up in bed 24 h after the operation, and to sit for half an hour every two hours, then slowly transition to being able to sit on the edge of the bed, and gradually try to stand up, and strive to get out of bed at 48 h after the operation. Oral nutrition was intensified, with patients transitioning from a liquid diet on the first day post-surgery, to a semi-liquid diet on the second day, and finally progressing to a normal diet on the third day after surgery.

### 2.6 Observations and follow-up

The patients were mainly observed for postoperative infections, wound dehiscence, hematoma, necrosis, and other complications and recurrence. Their basic preoperative information, operation time, postoperative first defecation time, first time of getting out of bed, drainage tube removal time, wound suture removal time, length of hospital stay, wound healing time, Vancouver Scar Scale, wound scar acceptance and VAS (postoperative day 1) were recorded. Follow-up procedures involved a combination of outpatient visits and telephone communication. Weekly follow-up appointments were scheduled after discharge until complete wound healing. Subsequent follow-up sessions occurred every three months for up to five years postoperatively.

The Ferriman-Gallway score, which uses a score for each of the 11 areas of the body, assigns a score of 0–4 to each area, with a total score of >6 for the 11 areas indicating excessive hair.

The Vancouver Scar Scale (VSS) is based on four aspects of scar color, vascular distribution, thickness, and softness; scores range from 0 to 15, with higher scores indicating more severe scarring and lower scores indicating milder scarring. To perform the assessment, a specialized slide is used to apply pressure to the scar for two seconds, followed by observation.

At 6 months postoperatively, patients rated the acceptability of the sacrococcygeal surgical scar on a scale ranging from 0 to 10. A score of 0 indicated complete unacceptability, while a score of 10 indicated complete acceptability.

The pain was scored according to the VAS, with a score of 0–10 according to the severity of the pain.

### 2.7 Statistical analysis

Statistical analyses were performed using SPSS 25.0 software. Statistically significant differences were determined by Student's *t* test or Chi-square test as appropriate. Probabilities of <0.05 were considered significant.

## 3 Results

### 3.1 All patients who meet the inclusion criteria

**Figure 7:**
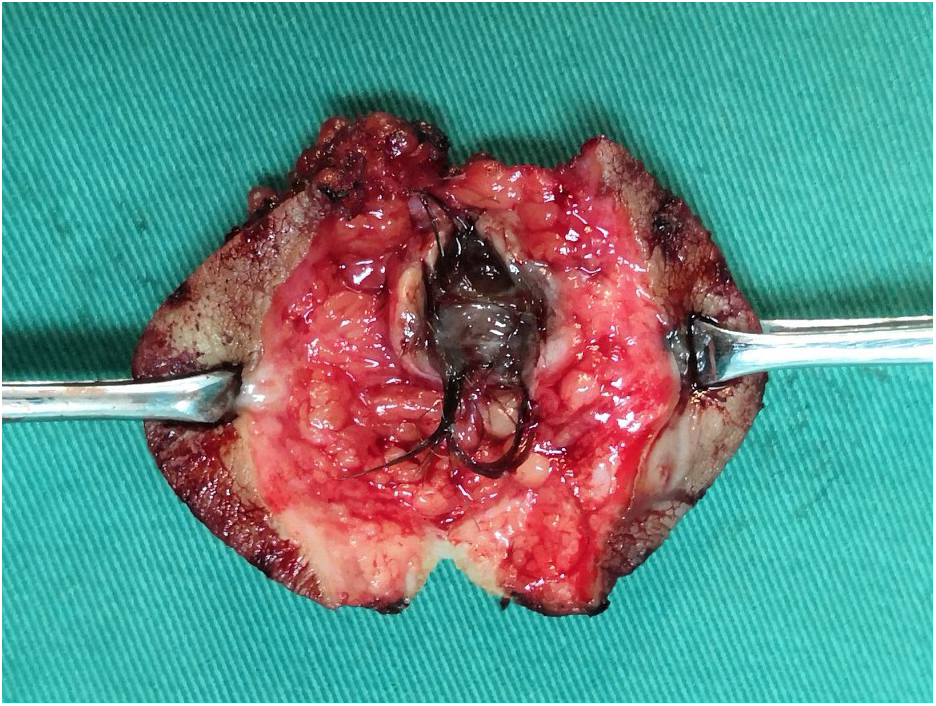
Hair was found in a resected specimen.

Among 109 patients who underwent surgical treatment meeting the inclusion criteria of the study, hair was found in 46 resected specimens (Figure 7). All surgical specimens were sent for pathological examination, and the results returned were consistent with chronic inflammatory-related changes in the pilonidal sinus. Comparing the control group with the observation group, the differences in the basic data of the patients (gender, age, BMI, duration of the disease, and Ferriman-Gallway score) were not statistically significant (*P* > 0.05) (Table 1).

**Table 1:** Comparison of basic information of patients in control group and observation group.

Group	Gender (M/F)	Age	BMI	Duration of disease (Month)	Ferriman-Gallway score
Control group (*n* = 58)	42/16	28.36 ± 7.66	26.20 ± 1.89	11.26 ± 6.98	12.72 ± 7.50
Observation group (*n* = 51)	38/13	27.53 ± 9.53	26.20 ± 1.66	11.51 ± 6.85	13.71 ± 6.96
*X* ^2^	0.06	–	–	–	–
*t*	–	0.51	−0.02	−0.19	−0.71
*P*	0.805	0.615	0.988	0.850	0.481

### 3.2 Comparison within the control group

Among patients who underwent pilonidal sinus resection with primary suture, the average operating time was 42.91 min. Twenty-eight patients received conventional postoperative rehabilitation (Control Group A), while thirty patients received ERAS (Control Group B). There were no significant differences in the baseline characteristics between the two groups. The median time to the first defecation was 7 days for Control Group A and 2 days for Control Group B. The median time to get out of bed was 8 days for Control Group A and 2 days for Control Group B. The median time for drainage removal was 8.5 days for Control Group A and 7 days for Control Group B. These differences were statistically significant. After combined ERAS, Control Group B was discharged earlier compared to Control Group A (9.00 ± 1.20 vs. 11.07 ± 1.78), and a significant difference was not observed in the time it took for wounds to heal (43.67 ± 7.26 vs. 41.64 ± 7.48); instead, there was a higher risk of wound dehiscence (26.7% vs. 7.1%), with 8 cases in Control Group B and only 2 cases in Control Group A (Table 2). The two groups did not exhibit differences in other complications, including infection, subcutaneous hematoma, ischemic necrosis, and the 5-year recurrence rate.

**Table 2:** Data were compared between patients in the control group who received conventional rehabilitation (control group A) and those who received ERAS (control group B).

Preoperative basic information									
Group		Gender (M/F)		Age	BMI	Duration of disease (month)		Ferriman-Gallway score	
Control group A (*n* = 28)		22/6		27.14 ± 8.86	26.45 ± 1.76	11.14 ± 8.30		14.25 ± 8.11	
Control group B (*n* = 30)		20/10		29.50 ± 6.30	25.96 ± 2.02	11.37 ± 5.61		11.30 ± 6.62	
*t*(*X*^2^)		1.03		−0.17	0.98	−0.12		1.52	
*P*		0.311		0.245	0.330	0.904		0.134	
Postoperative complications									
Group	VAS on the postperative day 1	Infection (%)		Dehiscence (%)	Subcutaneous hematoma (%)		Ischemic necrosis (%)		Recurrence (%)
Control group A (*n* = 28)	6.82 ± 0.61	6 (21.4)		2 (7.1)	9 (32.1)		0		7 (25.0)
Control group B (*n* = 30)	7.13 ± 0.78	7 (23.3)		8 (26.7)	8 (26.7)		0		9 (30.0)
*t*(*X*^2^)	−1.69	0.03		3.87	0.21		–		0.18
*P*	0.096	0.862		0.049	0.647		–		0.670
Operative time and postoperative recovery data									
Group	Operation time (min)	First defecation time (d)	First ambulation time (d)	Extubation time (d)	Stitch removal time (d)	Post-operative hospital stay (d)	Wound healing time (d)	Vancouver scar score	Wound scar acceptance
Control group A (*n* = 28)	42.50 ± 7.33	7.11 ± 0.92	8.04 ± 1.29	7.89 ± 1.10	14.75 ± 0.93	11.07 ± 1.78	41.64 ± 7.48	7.46 ± 1.77	7.61 ± 1.66
Control group B (*n* = 30)	43.30 ± 6.06	2.43 ± 0.50	2.30 ± 0.47	6.73 ± 0.58	14.40 ± 0.97	9.00 ± 1.20	43.67 ± 7.26	7.23 ± 1.36	7.73 ± 1.26
*t*	−0.45	23.83	22.21	5.19	1.40	5.15	−1.04	0.56	−0.33
*P*	0.651	<0.001	<0.001	<0.001	0.166	<0.001	0.300	0.745	0.868

### 3.3 Comparison within the observation group

A total of 51 patients underwent pilonidal sinus resection with Limberg flap graft with an average operative time of 78.16 min. 25 patients underwent conventional rehabilitation after surgery (Observation Group A) and 26 underwent ERAS (Observation Group B), with no significant difference in the basic data of the two groups. There were significant differences in the median times for the first defecation (7 days and 2 days), getting out of bed (8 days and 2 days), and drainage removal (8 days and 7 days). After combined ERAS, the discharge time of Observation Group B was earlier than that of Observation Group A (8.08 ± 1.20 vs. 9.16 ± 2.21), and the time of wound healing was much shorter (26.23 ± 3.97 vs. 29.08 ± 4.74), but there was no significant difference in the rate of postoperative complications between the two groups (Table 3).

**Table 3:** Data were compared between patients in the observation group who received conventional rehabilitation (observation group A) and those who received ERAS (observation group B).

Preoperative basic information									
Group	Gender (M/F)		Age		BMI		Duration of disease (month)	Ferriman-Gallway score	
Observation group A (*n* = 25)	20/5		27.16 ± 11.71		26.43 ± 1.29		12.00 ± 8.20	14.64 ± 7.42	
Observation group B(*n* = 26)	18/8		27.88 ± 7.06		25.98 ± 1.96		11.04 ± 5.36	12.81 ± .6.51	
*t*(*X*^2^)	0.78		−0.27		0.96		0.50	0.94	
*P*	0.378		0.789		0.340		0.621	0.353	
Postoperative complications									
Group	VAS on the postperative day 1	Infection (%)		Dehiscence (%)	Subcutaneous hematoma (%)		Ischemic necrosis (%)		Recurrence (%)
Observation group A (*n* = 25)	5.60 ± 0.96	3 (12.0)		0 (0.0)	0 (0.0)		5 (20.0)		2 (8.0)
Observation group B (*n* = 26)	5.00 ± 1.39	1 (3.8)		1 (3.8)	1 (3.8)		4 (15.4)		2 (7.7)
*t*(*X*^2^)	1.79	1.17		0.98	0.98		0.19		0.01
*P*	0.079	0.279		0.322	0.322		0.666		0.967
Operative time and postoperative recovery data									
Group	Operation time (min)	First defecation time (d)	First ambulation time (d)	Extubation time (d)	Stitch removal time (d)	Post-operative hospital stay (d)	Wound healing time (d)	Vancouver scar score	Wound scar acceptance
Observation group A (*n* = 25)	77.44 ± 8.65	6.68 ± 1.11	8.12 ± 1.17	7.92 ± 1.26	15.08 ± 1.00	9.16 ± 2.21	29.08 ± 4.74	7.40 ± 1.76	4.36 ± 0.91
Observation group B (*n* = 26)	78.85 ± 10.16	2.35 ± 0.49	2.27 ± 0.60	6.77 ± 0.71	14.38 ± 1.06	8.08 ± 1.20	26.23 ± 3.97	7.38 ± 1.33	4.00 ± 0.69
*t*	−0.53	17.98	22.63	4.05	2.41	2.16	2.33	0.04	1.59
*P*	0.598	<0.001	<0.001	<0.001	0.020	0.037	0.024	0.972	0.119

### 3.4 Comparison between control group B and observation group B

The data of patients receiving ERAS in the two groups with different surgical methods (Control Group B and Observation Group B) were compared, and no significant difference was found in the baseline characteristics between the two groups. The mean operation time of control group B was 43.30 min, while the mean operation time of observation group B was 78.85 min. The median time of first defecation, getting out of bed, drainage removal and stitches (2, 2, 7 and 14 days, respectively) were the same in the two groups. The mean postoperative pain score in Observation Group B was 5.00 and there was 1 case each of infection, laceration and hematoma, which were significantly less than Control Group B (7.13, 7, 8 and 8), but there were 4 cases of ischemic necrosis. The mean wound healing time in Observation Group B was 26.23 days, which was significantly less than that of Control Group B (43.67 days). After 5 years of follow-up, there were 2 recurrences in Observation Group B with a recurrence rate of 7.7%, and 9 recurrences in Control Group B with a recurrence rate of 30%. In Observation Group B, the mean Vancouver scar score was 7.38, and the mean surgical wound scar acceptance score was 4.00. In Control Group B, the mean Vancouver scar score was 7.73, and the mean surgical wound scar acceptance score was 7.53 (Table 4).

**Table 4:** Data on patients receiving rapid rehabilitation were compared between the control group and the observation group.

Preoperative basic information									
Group	Gender (M/F)		Age		BMI		Duration of disease (month)	Ferriman-Gallway score	
Control group B (*n* = 30)	20/10		29.50 ± 6.30		25.96 ± 2.02		11.37 ± 5.61	11.30 ± 6.61	
Observation group B (*n* = 26)	18/8		27.88 ± 7.06		25.98 ± 1.96		11.04 ± 5.36	12.81 ± 6.51	
*t*(*X*^2^)	0.04		0.91		−0.04		0.22	−0.86	
*P*	0.838		0.369		0.968		0.825	0.395	
Postoperative complications									
Group	VAS on the postperative day 1		Infection (%)	Dehiscence (%)		Subcutaneous hematoma (%)	Ischemic necrosis (%)		Recurrence (%)
Control group B (*n* = 30)	7.13 ± 0.78		7 (23.3)	8 (26.7)		8 (26.7)	0 (0.0)		9 (30.0)
Observation group B (*n* = 26)	5.00 ± 1.39		1 (3.8)	1 (3.8)		1 (3.8)	4 (15.4)		2 (7.7)
*t*(*X*^2^)	6.96		4.32	5.38		5.38	4.97		4.40
*P*	<0.001		0.038	0.020		0.020	0.026		0.036
Operative time and postoperative recovery data									
Group	Operation time (min)	First defecation time (d)	First ambulation time (d)	Extubation time (d)	Stitch removal time (d)	Post-operative hospital stay (d)	Wound healing time (d)	Vancouver scar score	Wound scar acceptance
Control group B (*n* = 30)	43.30 ± 6.06	2.43 ± 0.50	2.30 ± 0.47	6.73 ± 0.58	14.40 ± 0.97	9.00 ± 1.20	43.67 ± 7.26	7.73 ± 1.26	7.53 ± 0.86
Observation group B (*n* = 26)	78.85 ± 10.16	2.35 ± 0.49	2.27 ± 0.60	6.77 ± 0.71	14.38 ± 1.06	8.08 ± 1.20	26.23 ± 3.97	7.38 ± 1.33	4.00 ± 0.69
*t*	−15.60	0.66	0.22	−0.21	0.06	2.87	11.30	1.01	16.75
*P*	<0.001	0.514	0.831	0.836	0.955	0.006	<0.001	0.318	<0.001

## 4 Discussion

Research has shown that the overall satisfaction of patients after Limberg flap surgery is significantly higher than that of Karydakis flap surgery. In addition, the VAS score and incidence of postoperative complications of Limberg flap surgery were found to be lower than those of Karydakis flap surgery (30). Limberg flap surgery enables patients to resume daily activities earlier than V-Y flap surgery, with no significant difference observed in early complications between the two surgical procedures. However, after a follow-up of about 45 months, the recurrence rate of Limberg flap surgery was lower than that of V-Y flap surgery (1.5% vs. 11.1%) (31–33). Ray, K. et al. conducted and analyzed 18 RCTs involving 2,073 patients to compare the effectiveness of Limberg flap vs. Karydakis and/or Bascom procedure for the surgical excision of pilonidal sinus disease. The findings suggested that the Limberg flap exhibited a clinical advantage over the Karydakis and Bascom procedures in terms of reducing the recurrence rate following surgical excision of pilonidal sinus (34). The Z-plasty technique has been documented in numerous studies; however, overall wound complications and recurrence rates associated with this technique tend to be higher compared to other flap techniques (35). Therefore, in light of the aforementioned findings, the Limberg flap graft can generally be deemed superior to other flap techniques.

In comparison to pilonidal sinus resection with primary suture, this study suggests that while the wound associated with primary closure is less invasive and patients may find the postoperative scar more acceptable, it is more prone to infection and dehiscence. As such, the healing process is relatively slow, particularly among obese patients or those with larger lesions (36). Moreover, patients in the control group who received ERAS were more prone to experiencing wound dehiscence compared to those who received conventional postoperative rehabilitation. This suggests that perhaps the patients in the control group were not suitable candidates for ERAS. Pilonidal sinus resection with Limberg flap graft offers advantages such as reduced suture tension and shorter healing times. Further, after combining ERAS, there was an expedited discharge time, accelerated wound healing, and no significant increase in complications observed. However, the flap utilized in the procedure may risk ischemia, infection, and necrosis if the preoperative preparation is inadequate or if the intraoperative technique lacks precision. Thus, for acute infection of the pilonidal sinus, anti-infection treatment should be given first, and then surgery should be performed after the local inflammation has subsided. When making the flap, it is crucial to focus on safeguarding the flap vessels and ensuring that the flap's acute angle is not excessively small (<60°) (37). During suturing, it is essential for assistants to align and gently push the skin edges together to minimize tension and reduce the risk of wound tearing, bleeding, and other injuries. Intraoperatively, a negative pressure drainage tube is inserted to facilitate adequate drainage, with several lateral holes opened along the length of the drainage tube to ensure effective drainage. Postoperative prophylactic antibiotics are also routinely administered. Due to the large and deep nature of the wound, there is a possibility of a cavity forming beneath the suture site, which can lead to blood or fluid accumulation and increase the risk of infection. Therefore, early-stage pressure bandaging is necessary in conjunction with adequate drainage. Simultaneously, employing a flap technique can effectively disperse local tension and minimize the likelihood of wound rupture, thereby reducing complications such as bleeding and infection.

Moreover, patients undergoing primary suture may experience more intense postoperative pain at the suture site due to higher tension. Consequently, in conventional postoperative rehabilitation, patients may opt to remain in bed and limit movement due to the discomfort, which hinders early ambulation. Patients may develop a fear of eating due to concerns about subsequent defecation, resulting in reduced nutritional intake. This can consequently lead to delayed wound healing.

In the present study, the ERAS concept was integrated into both groups to enhance wound drainage, minimize infection risk, and facilitate wound recovery. This was achieved by enabling patients to commence an early high-protein diet and engage in appropriate activity soon after surgery. Observations were made that the patients in Group B had a median time to first defecation of 2 days after surgery, and a median time to first ambulation of 2 days after surgery, which was earlier than the median time to first defecation of 12 days and 8 days of ambulation after surgery reported in previous research (38). Previously, there was a belief that early ambulation after surgery might increase the risk of wound dehiscence and delay healing. Therefore, it was common practice to delay eating post-surgery, consequently delaying the first defecation. However, in the present study, it was found that in the control group, the incidence of most postoperative complications and wound healing time did not significantly differ in patients following ERAS compared to those adhering to fasting and strict bed rest. However, it was observed that the rate of wound dehiscence was higher in patients undergoing primary suture, which might be associated with early ambulation. As such, for patients in the control group, ERAS did not provide a significant benefit. In order to promote their wound recovery and reduce the risk of wound dehiscence, it may be necessary to delay the first feeding time or extend the liquid diet to delay the first defecation and thus delay ambulation. In the rehabilitation of some patients after Limberg flap graft, the ERAS concept was also implemented. These patients were encouraged to have their first meal on the first day after surgery. Notably, there was only one instance of wound dehiscence observed, which was possibly attributed to early defecation or ambulation. In addition, alternating between lying flat, lateral, and prone positions during bed rest may be beneficial for patients following Limberg flap graft surgery. This practice aims to prevent long-term pressure that could cause ischemic necrosis of the flap and alleviate discomfort associated with maintaining a single sleeping position for extended periods. Nonetheless, care should be taken to avoid increasing the sacrococcygeal tension when changing positions. For example, when transitioning a patient from a supine to a lateral position, the patient should align their torso along the longitudinal axis and turn onto their side with assistance from their own arms or with the help of others. During this process, it's important to ensure that the waist and hips remain relaxed to minimize strain. Additionally, providing support to the patient's back with pillows is advisable. When assisting the patient in getting out of bed, it is crucial to strictly prohibit any bending of the back and maintain a consistently straight posture of the waist and back. Using a bidet during toilet use is recommended to reduce tension on the lumbar and hip areas. For patients experiencing difficulty tolerating post-surgery wound pain, administering pain medication can help facilitate early movement out of bed. Surgeons opt for Limberg flap graft due to its low postoperative recurrence rate and broad applicability (31, 39, 40).

To further reduce the postoperative recurrence rate, it is also necessary to address the cause of the disease. The average age of the patients in the present study was 27.97 years old, primarily comprising young individuals whose occupations mainly included students and company employees. Given their predominantly sedentary lifestyles, there is an increased risk of frictional damage to the buttocks, making them susceptible to hair invasion. Therefore, it is recommended that patients reduce their sedentary time after discharge. For example, they should aim to stand up and move around for at least 15 min after sitting for an hour. The average BMI of all patients was 26.20, indicating that they were overweight on average. In overweight or obese individuals, deeper hip grooves and increased hair accumulation are common. Additionally, sedentary behavior in this population can exacerbate squeezing and frictional forces on the hips. Therefore, it is important for these patients to actively pursue weight reduction strategies after surgery. The author also found that 74 cases (including 4 females) exhibited increased hairiness in the sacrococcygeal region during patient examinations, with Ferriman-Gallwey scores ranging from 7 to 34, indicating excessive hair growth. As such, postoperative patients require regular hair removal to mitigate hair accumulation. Laser hair removal is recommended due to the risk of skin damage associated with shaving tools, which can increase the likelihood of hair penetration beneath the skin and induce pilonidal sinus recurrence. Research has shown that razor depilation can double the recurrence rate of pilonidal sinus (41).

## 4 Conclusion

The belief of the present authors is that pilonidal sinus resection with primary suture is simple and can be used for small lesions without obvious tension in the pilonidal sinus. In contrast, pilonidal sinus resection with Limberg flap graft is well-suited for all types of pilonidal sinuses, particularly when dealing with large lesions and evident tension in the area. Moreover, it boasts a low postoperative recurrence rate and fewer complications. This technique aligns well with the ERAS concept, facilitating a quicker return to normal activities such as work and study. Therefore, the combination of Limberg flap with ERAS holds significant value in pilonidal sinus treatment. It not only enhances surgical outcomes but also expedites patient recovery, making it a promising approach worthy of promotion and application.

## Data Availability

The original contributions presented in the study are included in the article/Supplementary Material, further inquiries can be directed to the corresponding authors.
